# Analysis of the Internal Bed Regulation Committees from hospitals of a Southern Brazilian city

**DOI:** 10.1590/S1679-45082017GS3878

**Published:** 2017

**Authors:** Vinícius Sabedot Soares

**Affiliations:** 1 Hospital Restinga e Extremo-Sul, Porto Alegre, RS, Brazil.

**Keywords:** Hospital administration, Bed occupancy, Health services accessibility, Health care coordination and monitoring, Health status indicators, Administração hospitalar, Ocupação de leitos, Acesso aos serviços de saúde, Regulação e fiscalização em saúde, Indicadores básicos de saúde

## Abstract

**Objective:**

To evaluate the composition of the Internal Regulation Committees created in hospitals of a capital city.

**Methods:**

A cross-sectional descriptive study assessing the structure, processes and results of each Committee.

**Results:**

The main reasons for implementing the committees were legal issues and overcrowding in the emergency department. The most monitored indicators were the occupancy rate and the mean length of stay, and the most observed results were reductions in the latter. Institutional protocols were developed in 70% of cases, and the degree of support that the Internal Regulation Committee received from the hospital managers was high, despite being only average the support received from the medical teams. Promoting the efficient use of beds seemed to be the main goal. To achieve it, the Internal Regulation Committee had to control hospital capacity at levels that allowed proper and safe bed turnover for patients. The strategies for this were varied and needed to integrate administrative and care issues.

**Conclusion:**

The Internal Regulation Committees were a management tool with great potential and promising results in the experiences evaluated.

## RESUMO

**Objetivo:**

Avaliar a composição dos Núcleos Internos de Regulação instituídos em hospitais de uma capital.

**Métodos:**

Estudo transversal e descritivo, com avaliação da estrutura, processos e resultados de cada núcleo.

**Resultados:**

As principais motivações para a criação dos núcleos foram as questões legais e a superlotação no setor da emergência. Os indicadores mais monitorados foram a taxa de ocupação e o tempo médio de permanência, e os resultados mais observados foram reduções neste último. Protocolos institucionais foram desenvolvidos em 70% dos casos, e o grau de suporte que o Núcleo Interno de Regulação recebeu da direção do hospital foi alto, apesar de ter sido apenas médio o suporte recebido das equipes médico-assistenciais. Promover o uso eficiente dos leitos pareceu ser o objetivo principal. Para atingi-lo, o Núcleo Interno de Regulação precisou controlar a lotação hospitalar em níveis que permitissem giro de leitos adequado e seguro para os pacientes. As estratégias para tal foram variadas e precisaram integrar as questões administrativas com as assistenciais.

**Conclusão:**

Os Núcleos Internos de Regulação foram uma ferramenta gerencial com grande potencial e resultados promissores nas experiências avaliadas.

## INTRODUCTION

Efficient management of the existing capacity has increasingly become a strategic issue in overcoming hospital overcrowding, a problem seen in many countries throughout the world. In Brazil, patients of the National Unified Healthcare System (SUS - *Sistema Único de Saúde*) have 11,938 fewer beds in the public hospital network, considering the period from 2008 to 2013.^(^ ^1^ ^)^

According to data from the Brazilian Institute of Geography and Statistics (IBGE - *Instituto Brasileiro de Geografia e Estatística*), the population segment that most increases in the Brazilian population is that of elderly individuals, with a growth estimate of more than 4% a year during the period from 2012 to 2022, and should reach 41.5 million people in 2030.^(^ ^2^ ^)^ The elderly epidemiological profile is very different from that of young people and adults, requiring more resources and a higher level of hospital use.^(^ ^3^ ^)^ Nevertheless, parallel to this, no increase is seen in available hospital beds.

In December 2013, the National Policy on Hospital Care (PNHOSP) was established by means of an Ordinance of the Ministry of Health.^(^ ^4^ ^)^ It provides the guidelines for the organization of the hospital component of the Healthcare Network (RAS - *Rede de Atenção à Saúde*). Among these recommendations, the creation of Internal Regulation Committees (NIR - *Núcleos Internos de Regulação*) in the hospital stands out: managerial structures of the existing capacity with the objective of organizing access to appointments, diagnostic and therapeutic services, and especially, to hospital beds. No formal implementation of standardization was established, and the hospitals that developed a NIR did so empirically, based on the particular needs of each organization.

## OBJECTIVE

To evaluate the Internal Regulation Committees created in hospitals of a capital city.

## METHODS

The composition of the NIR in the hospitals of Porto Alegre (RS) was assessed in this descriptive and comparative cross-sectional study, based on the information provided by their local coordinators. An online form was made available via internet, with specific multiple choice questions and open-ended essay questions. The information analyzed reflects the perception of the coordinators of each NIR, reported during the period of May 31, 2016 to June 10, 2016. The three spheres of quality in healthcare were observed (structure, processes, and results), as per the model proposed by Avedis Donabedian.^(^ ^5^ ^)^

## RESULTS

Porto Alegre is a city located in the southern region of Brazil, capital of the State of Rio Grande do Sul. It has a population of 1,476,867 inhabitants according to estimates for the year 2015.^(^ ^6^ ^)^ According to the National Registry of Healthcare Facilities (CNES - *Cadastro Nacional de Estabelecimentos de Saúde*), in May 2016, the city had 6,933 registered hospitalization beds in the clinical, surgical, and psychiatric areas, and 1,140 supplementary beds in Intensive Care Units (ICU) and Stepdown Units*.* This represents an overall rate of 54 beds per 10,000 inhabitants, or of 34 beds if we consider only the beds destined to the public system - SUS. The Brazilian average is 25 beds per 10 thousand inhabitants, while the worldwide average is 30 beds; comparatively, we can cite Germany with 82 beds, and the United States with 30 beds for every 10 thousand inhabitants.^(^ ^1^ ^)^

The hospital capacity of Porto Alegre is distributed over 27 institutions. Only 40% of the locations currently have a functioning NIR. The beds destined to SUS are regulated by a Municipal Hospital Bed Center, linked to the Municipal Secretariat of Health*.* This committee receives the transfer requests from the healthcare units of the network with a smaller diagnostic and therapeutic capacity, providing the search for beds for patients in the medium and high complexity organizations.

**Figure 1:**
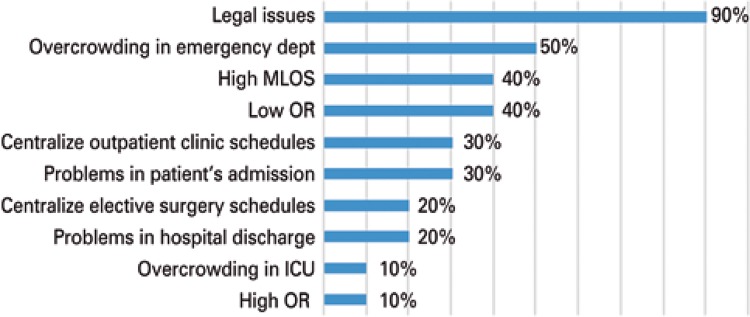
Motivations for the implementation of an Internal Regulation Committee

MLOS: mean length of stay in hospital; OR: occupancy rate; ICU: intensive therapy unit.

All the existing committees are in hospitals that have some bed destined to SUS patients (11 of 20 organizations). Of these, ten answered the evaluation form of this study. Only one NIR did not respond to the survey. The largest number of committees was created as of 2014 (seven of the ten evaluated) and among all of them, the legal issue related to the requirement of the PNHOSP Ordinance appears as the primary motivating factor for its implementation. Soon afterwards, the problem of overcrowding in the emergency department appears, besides the low occupation rate, with idleness of the existing capacity and prolonged hospitalization, along with long average hospital stays (Figure 1).

### Structure assessment

The number of professionals working at the NIR shows a great variation among the hospitals, considering that in 40% of cases, there are more than ten collaborators allocated, and the most common scenario is the presence of one physician and one nurse on the team; these are also the ones most frequently working exclusively at the NIR. In 60% of teams, the work is carried out from Monday to Friday, from 8:00am to 6:00pm, while three centers are open 24 hours a day and seven days a week. The existence of bed managers (a professional that manages real-time bed distribution) is not common in the team;^(^ ^7^ ^)^ this person is present in only 40% of centers.

The NIR has an office with its own room in 80% of hospitals, and all have access to equipment adequate for performing their work routine. In the institutional organization chart, the NIR is linked to the (medical) technical board in 60% of cases. In 90% of them, their scope of action is documented and validated by the board.

**Figure 2:**
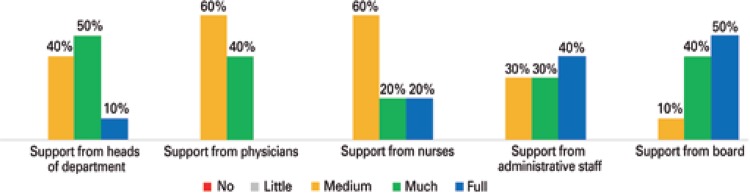
Level of support of the teams and of the direction for the execution of the work by the Internal Regulatory Committees

The collaboration of various sectors of the hospital, by means of the medical teams, nurses, and administrative staff, is fundamental for the execution of the NIR’s work (Figure 2).

### Process assessment

**Figure 3:**
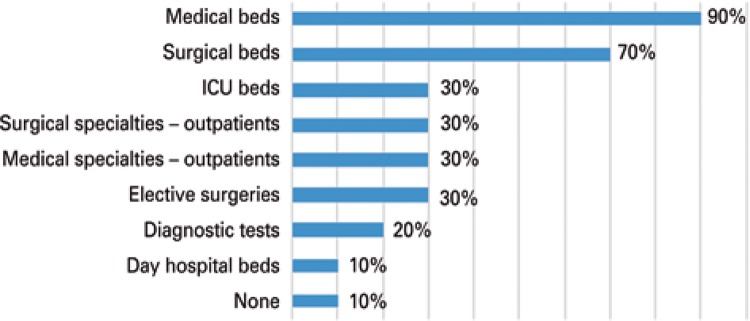
Frequency of access regulation by the Internal Regulatory Committee for hospital capacity resources

As to the routines, different compositions were noted for different diagnostic and therapeutic resources, and access to them is regulated directly by NIR. The components of the existing capacity most frequently regulated by the NIR are the beds in the clinical and surgical wards (Figure 3).

ICU: intensive care unit.

Almost all the committees (90%) reported having updated and daily knowledge of the hospital bed census. Most of them (70%) reported having implemented institutional routines in the form of operational protocols. Among those that developed protocols, the most common type is related to the process of hospital admission, with admission criteria. Only one NIR developed a protocol related to the regulation of access to the operating room.

The NIR is present in the meetings of committees or working groups in 90% of cases. The most common are the working groups of the Municipal Secretariat of Health. In this sense, the Access and Hospital Quality Committee (NAQH), recommended in the same ordinance that introduced the NIR,^(^ ^4^ ^)^ is the specific committee most often cited. Systematic participation of NIR in the daily medical rounds is an infrequent practice, even for the round routine only among members of the NIR team – it occurs in only about 30% of groups.

Most committees developed external relation routines with the other organizations of the city. However, only 30% has a direct relation with the Primary Care Units. Daily communication with the Central Municipal Committee on Bed Regulations occurred in most of them, and the quality of relation with this committee was considered excellent in 40%, good in 40%, and medium in 20% of cases.

### Result assessment

**Figure 4:**
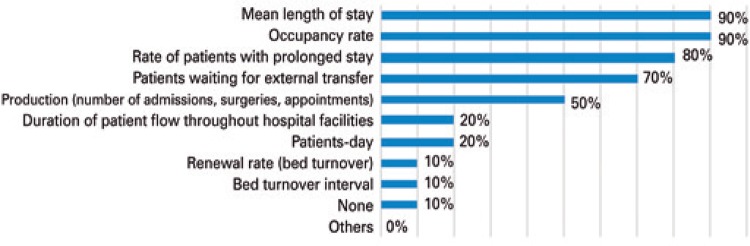
Frequency of indicators monitored by one of the Internal Regulatory Committees

The effectiveness of NIR work needs to be measured by means of indicators. There is variability in the composition of the list of indicators that are measured, as can be seen in figure 4. It is clear that the main objective of the NIR was to act in controlling the occupation rate and the average length of stay, indicators monitored in 90% of committees. Among the ten committees assessed, six declared having goals settled with the board of directors for an indicator.

In four out of ten institutions, changes were seen in the indicators after implementing the NIR. Of these, two reported that there was a reduction in the length of stay associated with raise in occupation rate; in the other two, reduction in mean length of stay or elevation of occupation rate, isolated, were observed.

## DISCUSSION

The theoretical concept of a NIR is comprehensive and includes the regulation of access to all diagnostic and therapeutic resources of the installed hospital capacity.^(^ ^4^ ^)^ What we noted in this survey was the greatest focus, often exclusive, on the regulation of access to beds in the clinical and surgical wards. Considering the overcrowding of the emergency department figures as the second motivating cause for the creation of a NIR, we came to understand this concern with the efficient use of the hospitalization beds.

The large number of cases seen in the emergency department of a hospital indicates low performance of the healthcare system, and the overcrowding of this unit reveals the low performance of the hospital and its network.^(^ ^8^ ^)^ Additionally, an overcrowded emergency department is an insecure environment for the patients, and is proven to be associated with worse clinical outcomes.^(^ ^9^ ^)^

As per the conceptual model of overcrowding in the emergency departments proposed by Asplin et al.,^(^ ^10^ ^)^ we can divide several causes of this problem into three time points of patient flow: input (demand, structure of the Primary Care network, etc.), throughput (reception, triage, consultation, tests, and treatment), and output (discharge, transfer, or hospital admission). According to a literature review, the increase in length of stay in the emergency department is the main marker of overcrowding and, and the lack of beds for hospitalization (output), its principal cause.^(^ ^9^ ^)^

The ineffective use of beds in sectors of the hospital leads to problems in the emergency department. It is necessary to recognize that its overcrowding is not a consequence merely of occasional dysfunctional routines of this department, but rather a reflection of the function of the entire hospital. The creation of a NIR has, therefore, as its primordial function to promote the efficient and rational use of the hospitalization beds. This should be done through actions that intend to reach results in the output stage of the emergency flow. This output stage is composed of three possibilities: discharge (with or without referral to the outpatient clinic), transfer to another organization, or hospital admission.

The admission of new patients depends on the existence of vacant beds and organized patient transport processes to these beds. Some studies have shown that hospital occupation rates over 85% compromise the dynamics of accommodation of new admissions via emergency sector, and when this indicator goes beyond 90%, the blockage of frequent access is expected, due to the lack of availability of free beds in the inpatients units.^(^ ^11^ ^,^ ^12^ ^)^

Maintaining the occupation rates within safe limits needs to be one of the primary goals of the NIR. Having a minimal supply of vacant beds allows the accommodation of variations in the demand for hospitalization, minimizing the risk of overcrowding crises in the emergency department. Nevertheless, curiously, four of the ten NIR evaluated in this study reported low occupation rates as one of the factors that motivated its creation; three of these were reference hospitals for clinical or surgical subspecialties, while one was a general hospital of intermediate complexity. After the creation of the NIR in these locations, the occupation rate increased.

It may seem a paradox that these organizations had been having idle beds in the current context of a large demand for few vacancies. This observed change leads one to conclude that it was necessary to effectively integrate these institutions into the existing capacity of the municipal SUS system. In this regard, it is likely that the establishment of a protocol with admission criteria, defining the profile of care, facilitated the allocation of the right patient to the right bed. Admission criteria can be developed taking into consideration which teams and structures will be available, as well as severity of the clinical condition is, which better indicates the location towards which to direct the patient within the organization − ward or ICU, for example.^(^ ^13^ ^)^ Even so, the strengthening of the external relations should have influenced the increase in patients referred to these hospitals.

The clarification of the care profile as per hospital admission institutional protocols helps the management of beds as well. This activity evolved to beyond the administrative and documental tasks associated with patient admission. In some hospitals, the scope was widened, with joint involvement of the medical teams.

Currently, the management of beds is considered an important tool for improvement of patient flow, and can be carried out by a nurse or physician (the “bed manager” or “bed czar”) with a strategic and operational character.^(^ ^14^ ^,^ ^15^ ^)^ The management of beds performed by this professional consists in organizing the allocation of new admissions to vacant beds, by means of real-time knowledge of the hospital census and of the demands for hospitalization. Additionally, the “bed manager” also evaluates and executes actions aiming to optimize the entire admission process until hospital discharge. In the ten committees assessed in this project, only four stated they had bed managers in their daily routine.

Still considering the expansion of NIR activities for the medical care sphere, there is room for participation in the daily discussions of the care teams, commonly called “rounds”. The performance of multidisciplinary rounds, in which planning for the hospital discharge is constant, proved effective in reducing the mean length of hospital stay.^(^ ^16^ ^)^ Among the committees of Porto Alegre, only 30% participated in this routine in the wards they regulated. To encourage the creation of these rounds in the teams, actively participating in them, is an opportunity to be considered by the NIR with the objective of reducing the mean length of stay.

Few NIRs (only 20% of them), among those that exist in the Porto Alegre hospitals, monitor the time of duration of the patient flow stages through the hospital structure. Several bottlenecks may exist in this flow, leading to delays in various stages of the entire process, increasing patient stay and contributing towards overcrowding.^(^ ^17^ ^,^ ^18^ ^)^ These flow inefficiencies can be evaluated and worked on according to different methodologies, and the one based on Lean principles – derived from a successful model of an automobile industry - has gained increasing prominence in the healthcare sector.^(^ ^19^ ^)^ The NIR needs to approve initiatives aiming to improve patient flow at the hospital, since the result should be promotion of efficiency in the use of the existing hospital capacity.

## CONCLUSION

The experience of the hospitals in Porto Alegre in the establishment of Internal Regulatory Committees is relatively recent, and most of them were constituted in the last two years. The management of access to the existing hospital capacity, in a broad sense, defines the general role of an Internal Regulatory Committee. In practice, we observe a greater focus on the regulation of access to clinical and surgical beds of the hospitals assessed.

The creation of an Internal Regulatory Committee has a large potential for the development of actions that result in efficient use of hospital capacity. In this regard, the dissemination of these committees in the hospitals that provide support for the Unified Healthcare System should be considered by the public administrators as a strategy for facing the lack of beds. The rational use of the hospital structure promoted by this committee also needs to be recognized by the organizations as fundamental for their sustainability, regardless of the market in which they work - whether public or private.
